# Splenic Infarction in Acute Cytomegalovirus and Epstein-Barr Virus Concomitant Infection

**DOI:** 10.7759/cureus.46235

**Published:** 2023-09-29

**Authors:** Tina Kana, Saraf Mehjabeen, Ahmed Kawamj, Nirav Patel, Zaineb Shamim

**Affiliations:** 1 Internal Medicine, Lenox Hill Hospital, New York City, USA; 2 Internal Medicine, Touro College of Osteopathic Medicine, New York City, USA; 3 Internal Medicine, New York Medical College, Passaic, USA; 4 Anesthesiology, Touro College of Osteopathic Medicine, Vallejo, USA; 5 Internal Medicine, NYU Langone, New York City, USA

**Keywords:** coinfection, splenomegaly, splenic infarction, ebv, cmv

## Abstract

In immunocompetent individuals, cytomegalovirus (CMV) infection can range from asymptomatic to infectious mononucleosis syndrome and can cause hemolysis. However, in immunocompromised individuals, the presentation may be complicated with various life-threatening complications. CMV-associated thrombosis is commonly reported in patients who are immunocompromised, especially in transplant recipients and in HIV-positive patients. We present a case of a previously healthy 29-year-old male patient who suffered a splenic infarction. He presented with high temperature, general malaise, and left-sided abdominal pain. He was diagnosed with CMV and Epstein-Barr virus concomitant infection. Serological studies confirmed an acute CMV infection superimposed on a chronic EBV infection.

## Introduction

Cytomegalovirus (CMV), officially known as HHV-5, belongs to the Herpesviridae family. In immunocompetent individuals, infections are generally asymptomatic and spread through oral secretions and body fluids. CMV infection is characterized by high fevers, myalgias, fatigue, sore throat, hepatosplenomegaly, and lymphadenopathy. The typical course of the virus is self-limiting in immunocompetent patients and rarely requires antiviral therapy. In immunocompromised patients, however, antiviral treatment is indicated and valganciclovir is the treatment of choice. Epstein-Barr virus, a double-stranded DNA virus known officially as HHV-4, belongs to the Herpesviridae family and is the most common cause of infectious mononucleosis worldwide with a prevalence rate of 90% [1]. A majority of cases are self-limiting and occur in children and adolescents. Symptoms include fever, lymphadenopathy, and fatigue; however, in rare cases, complications such as splenic rupture, hepatitis and severe tonsillar enlargement causing airway obstruction may develop [2]. A key distinction between the two viruses is with respect to where each establishes latency. CMV latency is maintained within blood monocytes and CD 34+ hematopoietic progenitor cells, while EBV primarily infects epithelial cells in the oropharynx and then replicates and spreads to B-cells resulting in latent infection [3]. Splenic infarction rarely occurs with infectious mononucleosis, and while splenic thrombosis is commonly associated with immunocompromised transplant patients and patients with HIV infection, we report a case of an immunocompetent patient with an acute CMV infection concomitant with a chronic EBV infection resulting in a spontaneous splenic infarction.

## Case presentation

A previously healthy 29-year-old male with morbid obesity with a BMI of 40.61 kg/m2, presented with a one-week history of left-sided abdominal pain and a six-day history of high-grade fever, with recorded daily temperatures between 101°F and 104.9°F associated with body aches, night sweats, and generalized weakness. The abdominal pain was rated 5/10 and did not radiate to the back or shoulder. The patient denied having any rashes, headaches, dizziness, changes in vision, sore throat, swollen glands, lumps in the neck, chest pain, shortness of breath, changes in bowel habits, palpitations, nausea, vomiting, numbness or tingling sensations. The patient had an appendectomy previously but otherwise had no significant medical or surgical history. He denied recent travel, and sick contacts and has been monogamous with one partner for the last seven years. The patient was diagnosed with COVID-19 in 2021 and has since been vaccinated. He also denied drug use and admitted to alcohol use socially. The patient did not have any children, nor did he have contact with children in his occupation. The patient's partner did not exhibit any symptoms during this time.

The patient's vital signs were a temperature of 100.3°F, a heart rate of 107 beats per minute, and a blood pressure of 112/63 mmHg. On physical examination, the head was normocephalic and atraumatic without tenderness, visible or palpable masses, depressions, or scarring. Visual acuity was intact and the conjunctivae were clear without exudates or hemorrhage. The sclera was non-icteric with extraocular movements intact. Pupils were equal, round, and reactive to light and accommodation. There were no signs of nystagmus. Examination of the ears revealed that the pinna, tragus, and ear canal were non-tender and without swelling. The tympanic membrane was normal in appearance with a good cone of light. The hearing was intact with good acuity to whispered voices. The nasal mucosa was pink and moist with the nasal septum being midline. The throat and mouth demonstrated pink and moist oral mucosa with good dentition. The tongue was normal in appearance without lesions and with good symmetrical movement. No buccal nodules or lesions were noted. Additionally, the pharynx was normal in appearance without tonsillar swelling or exudates. No adenopathy was noted. Cardiac auscultation revealed a regular S1 and S2 with no associated murmurs or rubs. Lung auscultation was unremarkable and an abdominal examination revealed a splenic tip close to the midepigastric area with no hepatomegaly. No lymph nodes were palpable in the groin, axilla, or neck with no swelling, asymmetry, discoloration, or increased temperature over lymph node locations.

There were no indications of lymphadenopathy or meningitis with no nuchal rigidity and a negative Kernig's and Brudzinski's sign. Initial blood tests demonstrated mildly elevated liver function tests with an aspartate transaminase (AST) level of 121 U/L, an alanine transaminase (ALT) level of 175 U/L, and a total bilirubin 1.4 mg/dL (Table 1). Alkaline phosphatase levels and white blood cell counts were within normal limits (Table 1). 

**Table 1:** Selected lab values BUN: blood urea nitrogen, AST: aspartate aminotransferase, ALT: alanine transaminase, GFR: glomerular filtration rate.

Test	Value	Reference Range
WBC	3.9 10*3/uL	3.5-10.5 10*3/uL
RBC	3.69 10*6/uL	3.9-5.03 10*6/uL
Hemoglobin	9.5 g/dL	12.0-15.5 g/dL
Hematocrit	28.70%	34.9-44.5%
Iron	44 ug/dL	60-170 ug/dL
Total Iron Binding Capacity	181 ug/dL	255-450 ug/dL
Iron Saturation	24.30%	20-50%
Ferritin	2070 ng/mL	12-300 ng/mL
Sodium	138 mmol/L	136-145 mmol/L
Potassium	4.6 mmol/L	3.5-5.3 mmol/L
BUN	6.4 mg/dL	6.0-24.0 mg/dL
Creatinine	0.7 mg/dL	0.5-1.0 mg/dL
Glucose	106 mg/dL	70-140 mg/dL
AST	121 U/L	10-36 U/L
ALT	175 U/L	6-29 U/L
Calcium	8.3 mg/dL	8.6-10.4 mg/dL
Sedimentation Rate	61 mm/hr	0-22 mm/hr
C-Reactive Protein	14.2 mg/L	< 10.0 mg/L
Vitamin B-12	639.8pg/mL	200-900 pg/mL
Dilute Russell's Viper Venom	42.4 seconds	35-45 seconds
Hepatitis A Ab, IgM	Negative	Negative
Hepatitis B Core Ab, IgM	Negative	Negative
Cytomegalovirus, IgM	>240 AU/mL	0-29.9 AU/mL
EBV Ab VCA, IgG	409 U/mL	0-18 U/mL
EBV Ab VCA, IgM	59.8 U/mL	0-36 U/mL
EBV Nuclear Antigen	223 U/mL	0-17.9 U/mL
Immunoglobulin A	186 g/L	0.8 - 3.0 g/L
Immunoglobulin G	1194 g/L	6.0 - 16.0 g/L
Immunoglobulin M	490 g/L	0.4 - 2.5g/L

**Figure 1:**
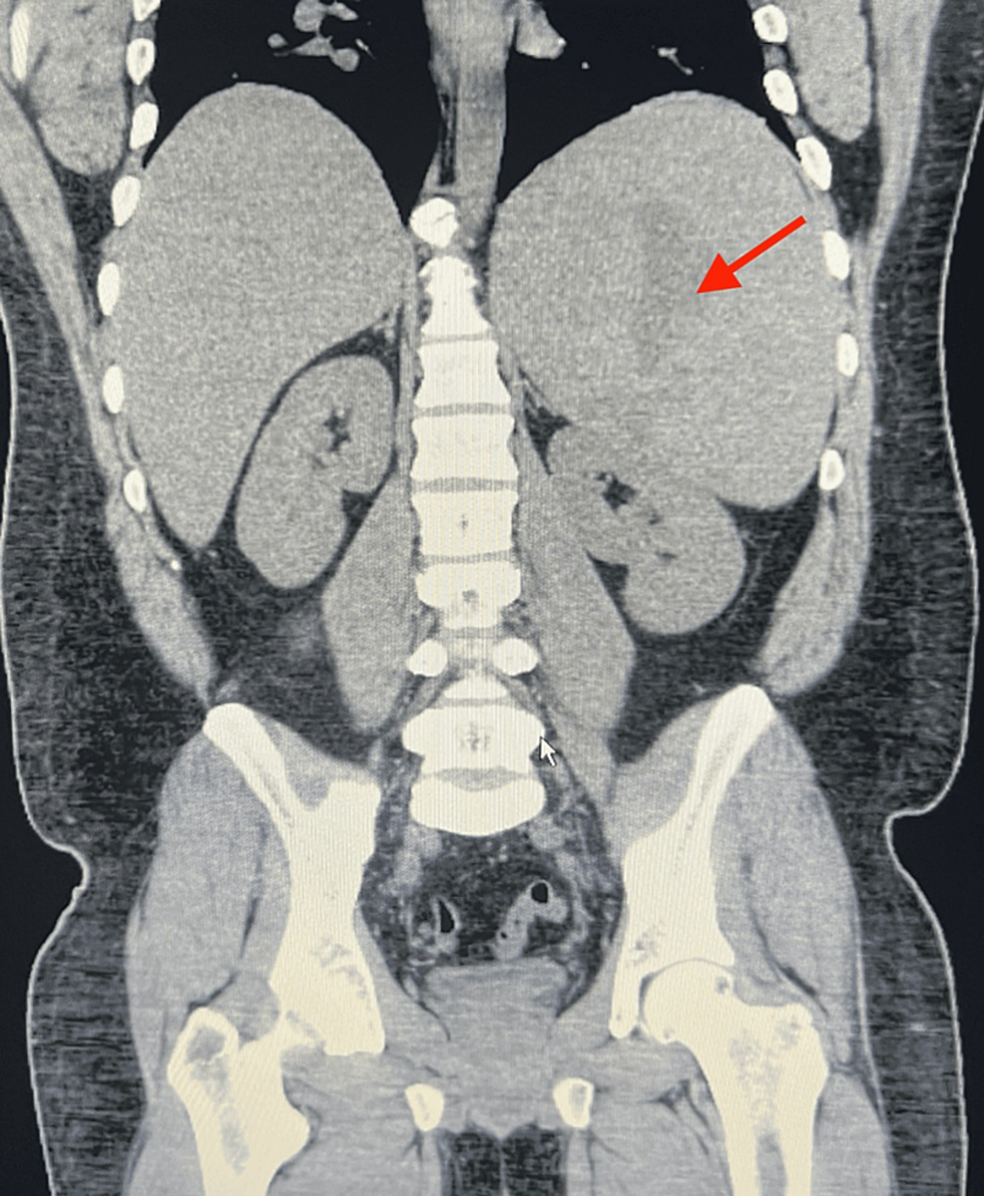
CT of the abdomen and pelvis showing marked splenomegaly

**Figure 2:**
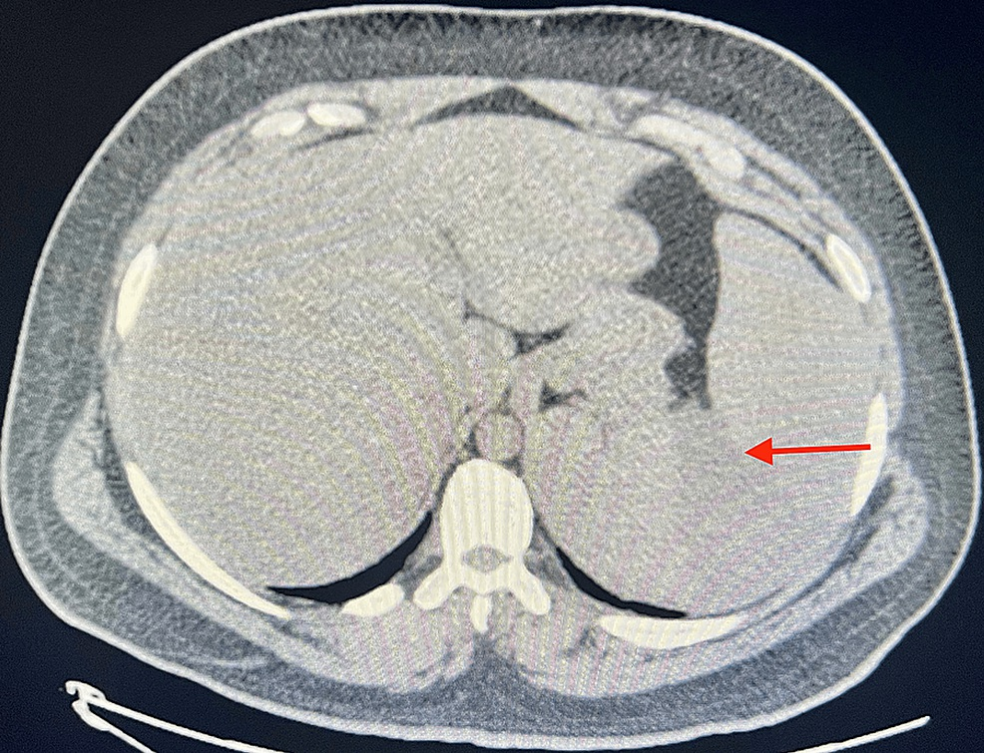
CT of the abdomen and pelvis showing splenic infarct

Echocardiography indicated a normal left ventricular ejection fraction of 60%-65% with no significant valvular regurgitation or stenosis and a chest x-ray demonstrated no dense consolidations or large effusions. Computed tomography with non-IV contrast of the abdomen and pelvis revealed marked splenic enlargement to 23 cm in length, with several splenic infarctions (Figures 1, 2). Additionally, the left kidney was slightly compressed by the enlarged spleen. Initial mono-spot testing was conducted which was non-reactive further testing with viral serology, and HIV testing yielded a negative result. The patient tested positive for CMV IgM antibodies with an IgM value of 240, indicating active infection, and had positive results for Epstein-Barr virus, with EBV IgG levels of 409 U/mL, IgM of 59.8 U/mL and EBV nuclear antigen of 223 U/mL (Table 1). A peripheral blood smear revealed a few enlarged cells with intranuclear cytoplasmic inclusions. Treatment was initiated with valacyclovir, prednisone, and enoxaparin. 

The patient continued to be febrile during his stay and developed night sweats, fatigue, and weakness secondary to the CMV infection. Symptoms associated with primary CMV infection can be prolonged, with fever lasting three weeks or more, while glandular fever typically lasts two to four weeks. Additional lab tests indicated elevated ESR and CRP levels. The patient was started on acetaminophen and empiric doxycycline. Over the next four days, the patient had significant improvement and was subsequently discharged on the fifth day of hospitalization. He was advised to strictly avoid all contact sports for the following six months due to the risk of splenic rupture. While typically asymptomatic and self-limiting, an acute CMV infection can demonstrate a rare propensity for vascular thrombosis, such as deep vein thrombosis, thrombophlebitis, and pulmonary embolism. Therefore, the decision was made to discharge the patient with acetaminophen, prednisone, valacyclovir, and apixaban for seven days. 

## Discussion

Splenic infarction, an extremely rare complication, occurs when splenic artery branches are obstructed by emboli or thrombi. Splenic infarction can result from a range of causes that include cardiovascular emboli, atrial fibrillation, and autoimmune disorders such as antiphospholipid syndrome. Additionally, infections with certain viruses that include HIV and varicella zoster virus have also been implicated in causing splenic infarctions due to induction of hypercoagulable states.

We report a case of a healthy young immunocompetent male with an acute CMV infection and a reactivated concomitant EBV infection who suffered from splenic infarctions. Complications of splenic infarctions due to acute CMV infection are extremely rare in immunocompetent individuals, and relatively few cases have been reported in the literature [4]. What remains unclear is the pathophysiological mechanism that explains exactly how CMV triggers thromboembolisms. Several theories have been proposed that CMV induces thromboembolism, by activating platelet and leukocyte adhesion to infected endothelial cells or by increasing levels of factor VIII and vascular smooth muscle proliferation [5]. CMV has been shown to increase platelet-derived growth factor (PDGF) and transforming growth factor-β, which can cause vascular cell wall proliferation. The most widely accepted theory describes a transient increase in anti-phospholipid antibodies due to CMV infection [6]. The patient in our case had a negative dilute Russell’s viper venom time with a result of 42.4. It has also been demonstrated that a CMV infection can cause activation of vascular cells leading to the expression of adhesion proteins thereby allowing platelets and leukocytes to adhere to the vascular wall, inducing a pro-inflammatory effect. This in turn promotes a hypercoagulable state [7]. The patient in our present case had an acute CMV infection superimposed on a reactivated latent EBV infection, which most likely resulted in a vascular environment demonstrating a greater change in coagulation. Regardless of the pathophysiological mechanism, there is increasing concern that CMV infections can induce vascular damage and thromboembolisms that can lead to life-threatening complications.

EBV, with a positive heterophile antibody test, is the virus that primarily causes Infectious mononucleosis, but other viruses such as CMV, parvovirus B19, HHV6, and HIV have been associated with infectious-mononucleosis-like syndromes with atypical lymphocytes [8]. The most widely used diagnostic criteria for infectious mononucleosis is Hoagland criteria, which states that for patients that present with clinical symptoms that include fever, pharyngitis and adenopathy with 50% lymphocytes and 10% atypical lymphocytes, the diagnosis should be confirmed by a positive serologic test for EBV [9]. Although our patient presented with high fever, splenomegaly, detected viral antigen and positive IgM titers to CMV and EBV, atypical lymphocytes were only 1.3%. Splenic infarction due to co-infection of CMV and EBV is very rare. In a 10-year retrospective case series study, only 49 patients out of 60,000 developed splenic infarctions, and only two of the 49 were diagnosed with infectious mononucleosis caused by an EBV infection [10]. 

Since viral infections were the root cause of splenic infarction, the patient was started on the antiviral medication, valacyclovir which resulted in improvement of the patient’s condition. CMV has a generally a self-limited course; however, this patient had a continuous fever for one week as well as splenomegaly with a splenic infarction, and the decision was made to initiate treatment based on the current literature. There are several published case reports and reviews that highlight the significant mortality and morbidity associated with untreated CMV infection in immunocompetent patients with rapid clinical improvement after anti-CMV therapy and steriod treatment [11]. The patient had a concomitant infection with CMV and reactivated EBV complicated by splenic infarction, in which antiphospholipid antibodies were not detected. Physicians should be aware of possible thromboembolisms, particularly in cases where patients are infected with more than one virus, during the acute phase of these infections.

## Conclusions

CMV is a common viral infection that often takes an asymptomatic or subclinical course in the immunocompetent patient. While syndromes like mononucleosis are the most prevalent presentation in this patient population, serious complications are a major concern in patients with an immunocompromised state. CMV-associated thrombosis is one such complication, where the literature pinpointing the exact pathophysiological mechanism remains unclear. We outline in this report, the case of a 29-year-old male suffering from a splenic infarct resulting from a CMV infection, secondary to a reactivation of an EBV infection. Given the broad prevalence of the virus and the fulminant nature of the more serious CMV complications, taking it into account early in the diagnostic process may facilitate treatment prior to the onset of such symptoms. Furthermore, more research is warranted to highlight the pathophysiology leading to these outcomes. 
